# The green tea extract epigallocatechin-3-gallate inhibits irradiation-induced pulmonary fibrosis in adult rats

**DOI:** 10.3892/ijmm.2014.1745

**Published:** 2014-04-16

**Authors:** HUA YOU, LI WEI, WAN-LIANG SUN, LEI WANG, ZAI-LIANG YANG, YUAN LIU, KE ZHENG, YING WANG, WEI-JING ZHANG

**Affiliations:** 1 Affiliated Hospital of the Academy of Military Medical Sciences, Beijing 100071, P.R. China; 2 Key Laboratory of Birth Defects and Reproductive Health of the National Health and Family Commission, Chongqing Population and the Family Planning Science and Technology Research Institute, Chongqing 400020, P.R. China; 3 Department of Cardiology, Cardiovascular Research Institute, Renmin Hospital, Wuhan University, Wuhan 430060, P.R. China; 4 Research Institute of Surgery, Daping Hospital, Third Military Medical University, Chongqing 400042, P.R. China; 5 Department of Endocrine Surgery, the First Affiliated Hospital of Chongqing Medical University, Chongqing 400016, P.R. China; 6 Clinical Laboratory, Beijing Chao-Yang Hospital, Capital Medical University, Beijing100020, P.R. China

**Keywords:** epigallocatechin-3-gallate, irradiation pulmonary fibrosis, alveolar epithelial type II cell, superoxide dismutase, nuclear transcription factor NF-E2-related factor 2

## Abstract

The present study evaluated the effect of epigallocatechin-3-gallate (EGCG), the most abundant catechin in green tea, on irradiation-induced pulmonary fibrosis and elucidated its mechanism of action. A rat model of irradiation-induced pulmonary fibrosis was generated using a ^60^Co irradiator and a dose of 22 Gy. Rats were intraperitoneally injected with EGCG (25 mg/kg) or dexamethasone (DEX; 5 mg/kg) daily for 30 days. Mortality rates and lung index values were calculated. The severity of fibrosis was evaluated by assaying the hydroxyproline (Hyp) contents of pulmonary and lung tissue sections post-irradiation. Alveolitis and fibrosis scores were obtained from semi-quantitative analyses of hematoxylin and eosin (H&E) and Masson’s trichrome lung section staining, respectively. The serum levels of transforming growth factor β1 (TGF-β1), interleukin (IL)-6, IL-10, and tumor necrosis factor-α (TNF-α) were also measured. Surfactant protein-B (SPB) and α-SMA expression patterns were evaluated using immunohistochemistry, and the protein levels of nuclear transcription factor NF-E2-related factor 2 (Nrf-2) and its associated antioxidant enzymes heme oxygenase-1 enzyme (HO-1) and NAD(P)H:quinone oxidoreductase-1 (NQO-1) were examined via western blot analysis. Treatment with EGCG, but not DEX, reduced mortality rates and lung index scores, improved histological changes in the lung, reduced collagen depositions, reduced MDA content, enhanced SOD activity, inhibited (myo)fibroblast proliferation, protected alveolar epithelial type II (AE2) cells, and regulated serum levels of TGF-β1, IL-6, IL-10, and TNF-α. Treatment with EGCG, but not DEX, activated Nrf-2 and its downstream antioxidant enzymes HO-1 and NQO-1. Taken together, these results showed that EGCG treatment significantly inhibits irradiation-induced pulmonary fibrosis. Furthermore, the results suggested promising clinical EGCG therapies to treat this disorder.

## Introduction

Irradiation-induced pulmonary fibrosis is a major complication associated with total body irradiation for hematopoietic stem cell transplantation, nuclear accidents, and thoracic radiotherapy for lung cancer, breast cancer, thymoma, and lymphoma (1,2). This complication develops ~6 months to several years after radiation exposure in humans and 100–120 days post-irradiation in the C57BL/6J mouse model (2). Recent clinical data have demonstrated that the incidence of irradiation-related pulmonary injury among patients with cancer who received radiotherapy ranged from 20.3% to 36.9% (3–6). The current clinical treatment for pulmonary fibrosis primarily involves drugs such as steroids or non-steroidal anti-inflammatory agents and immunosuppressive agents. These drugs can decrease acute pneumonitis for 2–3 months after irradiation, but they cannot effectively mitigate fibrosis (2). In addition, immunosuppressive agents can cause serious side-effects, including death.

Oxidative stress begins at radiation exposure and is sustained throughout the disease’s progression, presumably through the radiation-induced activation of oxidant-generating enzymes, mitochondrial leakage, and the activation of the respiratory burst in the phagocytic cells that infiltrate damaged tissue (7). Therefore, antioxidant treatment strategies are being developed to treat irradiation-induced pulmonary fibrosis. Epigallocatechin-3-gallate (EGCG) is a natural antioxidant derived from green tea that has attracted particular attention and recognition for its potential applications in the treatment of oxidative stress-related diseases including cancer, cardiovascular diseases, and neurodegenerative diseases (8). EGCG is the primary component of tea polyphenols, and it has shown a wide range of biological activities and pharmacological effects *in vitro* and *in vivo*, including antioxidant, anti-free radical, anti-mutagenic, and antitumor effects (9). EGCG inhibits chemical-induced lung fibrosis (10–13) and liver fibrosis (14). However, irradiation-induced pulmonary fibrosis and acute chemical-induced lung injury are not identical, particularly with respect to their pathogeneses. As yet, no study has examined the efficacy or mechanism of action of EGCG with regard to preventing or treating irradiation-induced pulmonary fibrosis.

To counteract the oxidative stress induced by reactive oxygen species (ROS), lung cells activate a wide variety of endogenous antioxidant enzymes including catalase, superoxide dismutases, and peroxiredoxins. The transcription of these cytoprotective enzymes is regulated by the nuclear transcription factor NF-E2-related factor 2 (Nrf2), which plays a central role in the regulation of cellular redox status. Under normal homeostatic conditions, Nrf2 transcription is repressed by its negative regulator Kelch-like ECH-associated protein 1 (Keap1). However, following exposure to ROS, Nrf2 dissociates from cytosolic Keap1 and translocates to the nucleus, where it binds to the antioxidant response element (ARE) in the promoter regions of the genes that encode antioxidant enzymes and induce their transcription (15). The antioxidant enzyme system regulated by the Nrf2-ARE signaling pathway is primarily composed of heme oxygenase-1 (HO-1), γ-glutamine cysteine synthetase (γ-GCS), NAD(P)H:quinone oxidoreductase-1 (NQO-1) and superoxide dismutase (SOD).

In the present study, we hypothesized that the administration of EGCG would significantly inhibit irradiation-induced pulmonary fibrosis. We first evaluated the efficacy of EGCG to ameliorate irradiation-induced pulmonary fibrosis and then examined whether EGCG treatment influenced Nrf-2, HO-1 and NQO-1 levels in irradiated rats. To the best of our knowledge, this study is the first to assess and highlight the efficacy of EGCG in the treatment of irradiation-induced pulmonary fibrosis.

## Materials and methods

### Animals

Male 6- to 8-week-old Sprague-Dawley (SD) rats (Laboratory Animal Center, Academy of Military Medical Sciences, Beijing, China) weighing 180–200 g were housed in an SPF-graded animal care facility according to the guidelines of the National Institutes of Health and Academy of Military Medical Sciences for the Care and Use of Laboratory Animals. The Committee on the Ethics of Animal Experiments of the Affiliated Hospital of Academy of Military Medical Sciences approved the protocol. All interventions were performed under sodium pentobarbital anesthesia, and all efforts were made to minimize suffering. Rats were provided with pathogen-free water and food for maintenance and caged in a controlled SPF environment with a 12/12-h light/dark cycle. Rats were observed daily up to 4 months post-irradiation, with particular attention afforded to difficulties in breathing, ruffling of the fur, hunched posture, and decreased breathing rate. The natural death of the animals was recorded.

### Irradiation and treatment

A ^60^Co irradiator [Reviss Services (UK), Ltd., Buckinghamshire, UK] was used to generate gamma-ray radiation. The rats were irradiated to 22 Gy at a dose rate of 290 cGy/min. The beam was restricted to the entire thorax. After anesthetization with 3% sodium pentobarbital (45 mg/kg) and irradiation as described above, the rats were intraperitoneally injected with EGCG (25 mg/kg; Sigma, St. Louis, MO, USA) (n=40) or dexamethasone (DEX; n=40; 5 mg/kg; Tianjing Pharmaceuticals Group Corp., Tianjing, China) daily for 30 days. Irradiated rats (radiation only; n=40) received radiation without treatment. The control group (n=40) was composed of normally fed, age-matched animals that were not irradiated. Similar doses of EGCG (25 mg/kg) were used in previous studies (11–13).

### Specimen processing and histopathology

After measuring body weight, six rats from each group were sacrificed at 15, 30, 60 or 120 days after initiation of the experiment. The wet weight of the lungs was recorded for each animal. The left lungs were frozen with dry ice powder and kept at −70°C for later use. The right lungs were fixed with 4% paraformaldehyde for histological and immunohistochemical analyses. Blood samples were collected from the heart and allowed to clot for 1 h at room temperature. Serum samples were obtained by centrifugation at 3,500 rpm for 5 min at 4°C and then stored at −70°C. The right lungs were dehydrated in ethanol and embedded in paraffin. Lung sections (5 μm) were stained with hematoxylin and eosin (H&E), Masson’s trichrome (Masson) and Sirius red.

### Lung index measurement

The ratio of the lung wet weight (mg) to body weight (g) was used as the lung index.

### Measurement of collagen content in the lungs

Lung collagen content was determined using the hydroxyproline (Hyp) assay according to the manufacturer’s protocol (Nanjing Jiancheng Bioengineering Institute, Nanjing, China). Approximately 100 mg of left lung tissue was hydrolyzed in 1 ml of lysis buffer solution at 100°C for 20 min. The absorbance of colored products was measured at 550 nm.

### Malondialdehyde (MDA) content and SOD activity measurements in serum

Serum samples were assayed for MDA content and SOD activity using commercially available kits according to the manufacturer’s instructions (Nanjing Jiancheng Bioengineering Institute). MDA, an end product of ROS-induced peroxidation of cell membrane lipids, is a reliable marker of oxidative damage (16). MDA content was determined by measuring chromogen generation from the reaction of MDA with 2-thiobarbituric acid. SOD activity was measured by monitoring the sample’s capacity to inhibit the reduction of ferricytochrome *c* via xanthine/xanthine oxidase. Briefly, this method is dependent on the inhibition of nitroblue tetrazolium (NBT) reduction via the xanthine/xanthine oxidase system as a superoxide generator (17).

### Immunohistochemical analyses

Lung sections (5 μm) were deparaffinized, rehydrated through a graded alcohol series, and exposed to a microwave-based antigen retrieval with a citrate buffer (10 mM of sodium citrate, pH 6.0 for 15 min). Endogenous peroxidases were quenched using 3% H_2_O_2_ for 5 min. The sections were incubated with surfactant protein-B (SPB; 1:200) or α smooth muscle actin (α-SMA; 1:200) (both from Boster Biological Technology, Wuhan, China) antibodies at 37°C for 2 h. The primary antibody was omitted in the negative control samples. After washing with PBS, the sections were incubated with poly-peroxidase-conjugated anti-mouse/rabbit IgG for 30 min at 37°C using the Polymer-HRP Detection System (Zymed Laboratories, South San Francisco, CA, USA) according to the manufacturer’s instructions. The slides were visualized with diaminobenzidine (DAB; Dako, Glostrup, Denmark), counterstained with Mayer’s hematoxylin, dehydrated through increasing concentrations of alcohol, cleared in xylene, and mounted in neutral balsam (Sigma).

### Serum cytokine levels

Serum levels of TGF-β1 were determined using the commercially available TGF-β1 ELISA kit according to the manufacturer’s instructions (Boster Biological Technology). The OD value was determined at 450 nm using an ELISA reader and calculated at the linear portion of the curve. Serum levels of IL-6, IL-10, and TNF-α were measured using flow cytometric bead assays according to the manufacturer’s instructions (BD™ CBA Flex Set; BD, Sparks, MD, USA).

### Western blot analysis

Frozen left lungs were pulverized and lysed in RIPA buffer. Lysates were centrifuged at 12,000 rpm and 4°C for 10 min, and the supernatants were collected for total protein analysis. A BCA protein assay kit (Beyotime Institute of Biotechnology, Jiangsu, China) was used to determine protein concentrations. Equal amounts of protein were separated by SDS-PAGE, transferred to a PVDF membrane (Millipore Corp., Billerica, MA, USA), and incubated with 5% BSA at room temperature for 2.5 h to block non-specific binding. The membranes were then incubated with the following primary antibodies at room temperature for 3 h: Nrf-2 (1:200; Sigma), HO-1 (1:200), NQO-1 (1:200) (both from Millipore) or β-actin (1:2,000; Cell Signaling Technology, Inc., Danvers, MA, USA). After washing with TBS-T, the membranes were incubated with HRP-conjugated secondary antibodies. The protein bands were visualized using enhanced chemiluminescence with a Super Signal detection kit (Boster Biological Technology).

### Morphometric analyses

Following the methodology described by Szapiel *et al* (18), lung sections stained with H&E or Masson’s trichrome were scored for alveolitis and fibrosis, respectively. Briefly, the severity of alveolitis and fibrosis was graded and scored on a scale of 0–3 (18): grade 0, normal lung; grade 1, minimal lesion (lesion area <20%); grade 2, moderate lesion (lesion area, 20–50%); or grade 3, severe lesion (lesion area >50%). Ten fields per section at ×100 magnification were randomly selected per rat, and two blinded pathologists carefully and independently examined 60 fields per group using an Olympus microscope (Olympus, Tokyo, Japan). The total score of each section was calculated, and the mean score of each group was determined as the total score of all sections divided by six. Lung sections stained with Sirius red were observed and images were captured using a polarizing microscope. For the immunohistochemical analyses of SPB and α-SMA, staining density was determined using Image Proplus software in one field with a prominent DAB reaction for each section under ×200 magnification for a total of six fields per group. Large airways and lung vessels were excluded from all analyses.

### Statistical analysis

Data were expressed as the means ± standard deviations (SDs). Between-group differences were tested using a two-way ANOVA followed by Tukey’s post-hoc test. Two-group comparisons were performed using independent-samples Student’s t-tests. P<0.05 was considered significant.

## Results

### EGCG reduces mortality and inhibits the formation of fibrous nodules in pulmonary tissue

Irradiated rats treated with EGCG (17.5%, 7/40) had a lower mortality rate compared with irradiated rats treated with DEX (27.5%, 11/40) and those that received radiation only (25.0%, 10/40). All control animals survived to 4 months without death.

**Figure 1:**
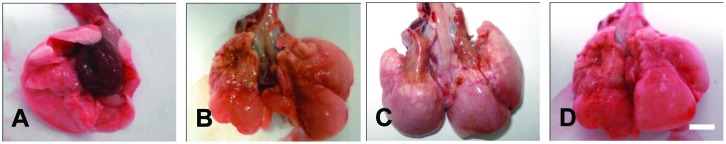
Effect of epigallocatechin-3-gallate (EGCG) on the lung appearance at 120 days post gamma-ray irradiation. (A) Hematoxylin and eosin (H&E) staining of lung tissue from non-irradiated normal control, (B) untreated irradiated, but (C) dexamethasone (DEX)-treated irradiated and (D) EGCG-treated irradiated rats. Untreated irradiated and DEX-treated irradiated animals show marked lung collapse, rough surfaces and gray fibrous nodule development. EGCG treatment animals show lung tissue edema, rough surfaces, scattered punctate bleeding, but no lung collapse and gray fibrous nodules. Bar, 0.5 cm.

Congested edema and bleeding sites were significantly attenuated in DEX-treated pulmonary tissues compared with radiation-only animals at 15 and 30 days post-irradiation. Pulmonary collapse and gray fibrous nodules were similar in DEX-treated and radiation-only animals at 60 and 120 days post-irradiation. Bleeding sites were seldom observed in EGCG-treated pulmonary tissues at 15 days post-irradiation. Signs of congested edema were also significantly attenuated in EGCG-treated pulmonary tissues compared with DEX-treated and radiation-only tissues at 15, 30 and 60 days post-irradiation. At 120 days post-irradiation, the lungs of EGCG-treated rats showed signs of edema and uneven surfaces as well as scattered punctuate bleeding points. However, neither pulmonary collapse nor gray fibrous nodules were found in EGCG-treated animals at 120 days post-irradiation (Fig. 1). These results suggested that EGCG significantly ameliorates irradiation-induced pulmonary fibrosis.

### EGCG reduces the lung index score

**Figure 2:**
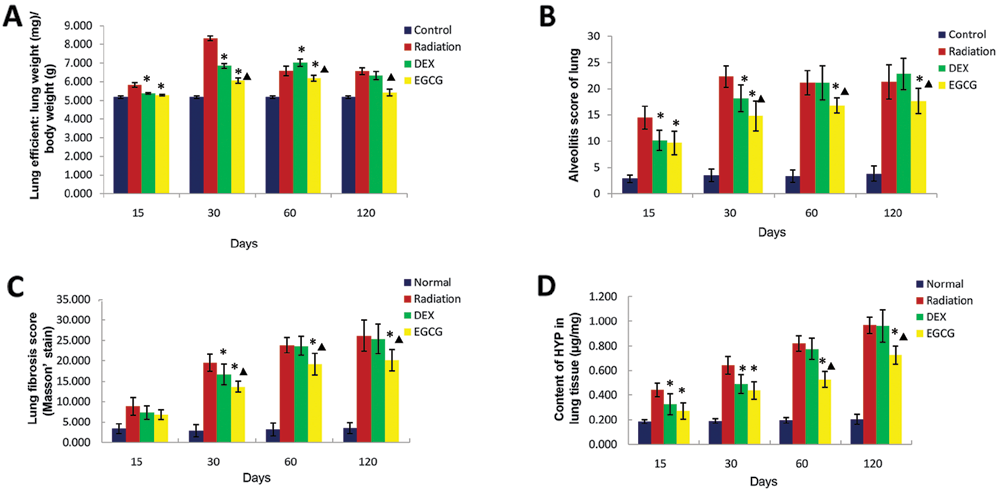
(A) The effect of epigallocatechin-3-gallate (EGCG) on the lung index score, (B) combined alveolitis score, (C) combined fibrosis score, and (D) hydroxyproline (Hyp) content at 15, 30, 60 and 120 days post-irradiation. (A) Lung index values were significantly lower (p<0.05) among the EGCG-treated animals compared with those of the DEX-treated animals at 30 and 0 days post-irradiation but significantly higher (p<0.05) at 120 days post-irradiation. Lung index values were significantly lower (p<0.05) among dexamethasone (DEX)-treated animals compared with those of untreated animals at 15, 30 and 60 days post-irradiation but similar at 120 days post-irradiation. (B) The combined alveolitis score was significantly lower (p<0.05) among EGCG-treated animals than that among DEX-treated animals at 30, 60 and 120 days post-irradiation. This score was also significantly lower (p<0.05) among DEX-treated animals compared with that of untreated animals at 15 and 30 days post-irradiation but similar at 60 and 120 days post-irradiation. (C) The combined fibrosis score was significantly lower (p<0.05) among EGCG-treated animals compared with that of DEX-treated animals at 30, 60 and 120 days post-irradiation. The fibrosis score was significantly lower (p<0.05) among DEX-treated rats compared with that of untreated rats at 30 days post-irradiation but similar at 60 and 120 days post-irradiation. (D) The Hyp content was significantly lower (p<0.05) among EGCG-treated animals compared with that of DEX-treated animals at 60 and 120 days post-irradiation. This parameter was significantly lower (p<0.05) among the DEX-treated rats relative to that of the untreated animals at 15 and 30 days post-irradiation but similar at 60 and 120 days post-irradiation. The bars in the graph are the standard deviations (SDs). Asterisks show significance (p<0.05) compared with the untreated radiation-only rats, and stars show significance (p<0.05) compared with DEX-treated animals.

We examined whether EGCG treatment influenced the lung index, which refers to the ratio of lung wet weight to body weight. The lung index was significantly lower (p<0.05) in EGCG-treated animals relative to DEX-treated animals at 30 and 60 days post-irradiation but significantly higher (p<0.05) at 120 days post-irradiation. This result matches the morphometric observations of lung appearance in EGCG-treated animals at 120 days post-irradiation (Fig. 1). At this time point, edema was still detectable in EGCG-treated animals but was not detected in DEX-treated and untreated irradiated animals. The lung index score was significantly lower (p<0.05) among the DEX-treated animals relative to the radiation-only animals at 15, 30 and 60 days post-irradiation but similar at 120 days post-irradiation (p>0.05; Fig. 2A). These results indicated that EGCG significantly attenuated congested edema in pulmonary tissues post-irradiation.

### EGCG improves histological changes and reduces collagen deposition in pulmonary tissues

**Figure 3:**
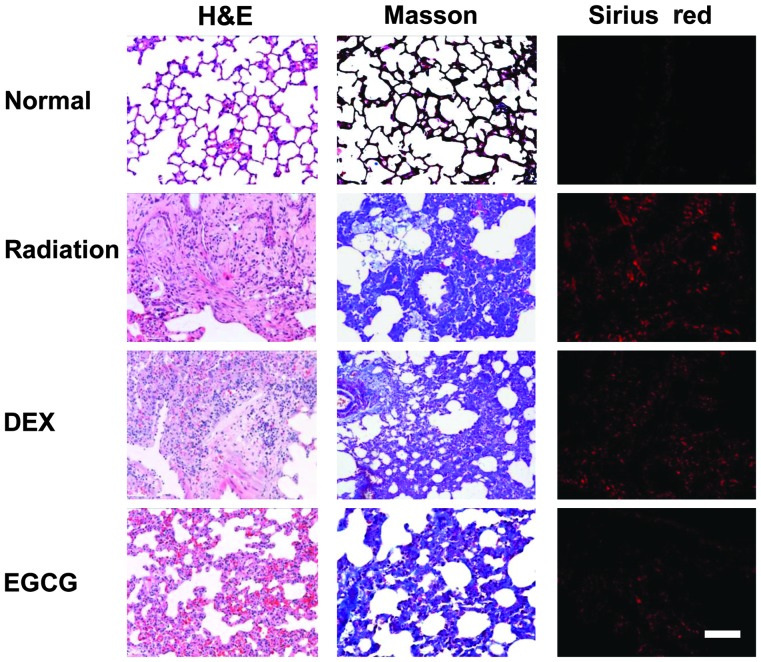
Effect of epigallocatechin-3-gallate (EGCG) on the histological changes in lung tissue at 120 days post-irradiation. Photomicrographs show staining of rat lung tissue sections with hematoxylin and eosin (H&E) (left column), Masson’s trichrome (middle column) and Sirius red (right column) from animals in the non-irradiated control (normal), irradiated but untreated (radiation) dexamethasone (DEX)-treated, and EGCG-treated (EGCG) groups. Note that inflammatory cell infiltration, fibrotic lesions and collagen fiber deposition were significantly improved in EGCG-treated animals. Bar, 100 μm.

We examined whether EGCG treatment improves the histological changes that occur in pulmonary tissues post-irradiation. We performed H&E, Masson’s trichrome and Sirius red staining of the lung sections to observe histological changes. A semi-quantitative analysis involving alveolitis and fibrosis scores was performed on the H&E- and Masson-stained sections following Szapiel’s method (18). Markedly thickened alveolar walls, collapsed alveoli, foam-like cells in the alveolar space, diffuse accumulations of inflammatory cells, extensive depositions of collagen, and regional fibrotic foci were observed in the H&E-stained irradiated pulmonary tissues at 120 days post-irradiation. Treatment with EGCG, but not DEX, significantly improved irradiation-induced pathological changes (Fig. 3, left column). Similarly, Masson’s trichrome (Fig. 3, middle column) and Sirius red staining (Fig. 3, right column) of the lung sections revealed that the regional fibrotic foci and collagen depositions were greatly reduced after EGCG treatment.

The alveolitis and fibrosis scores of EGCG-treated animals were significantly lower (p<0.05) than those of DEX-treated animals at 30, 60 and 120 days post-irradiation. The alveolitis score of the latter group was significantly lower (p<0.05) than that of the radiation-only animals at 15 and 30 days post-irradiation. The fibrosis score of DEX-treated animals was significantly lower (p<0.05) than that of radiation-only animals at 30 days post-irradiation. The alveolitis and fibrosis scores of DEX-treated animals were similar to those of radiation-only animals but significantly higher (p<0.05) than those of EGCG-treated animals at 60 and 120 days post-irradiation (Fig. 2B and C).

We also examined the degree to which EGCG treatment eliminated Hyp, the major constituent of collagen, from pulmonary tissues. The amount of Hyp was significantly lower (p<0.05) in EGCG-treated animals than DEX-treated animals at 60 and 120 days post-irradiation. The Hyp content of the latter group was significantly lower (p<0.05) than that of the radiation-only animals at 15 and 30 days post-irradiation but similar to that of these animals, and significantly higher (p<0.05) than that of the EGCG-treated animals, at 60 and 120 days post-irradiation. Together, these results showed marked anti-fibrotic effects of EGCG *in vivo* (Fig. 2D).

### EGCG modulates the serum redox state

**Figure 4:**
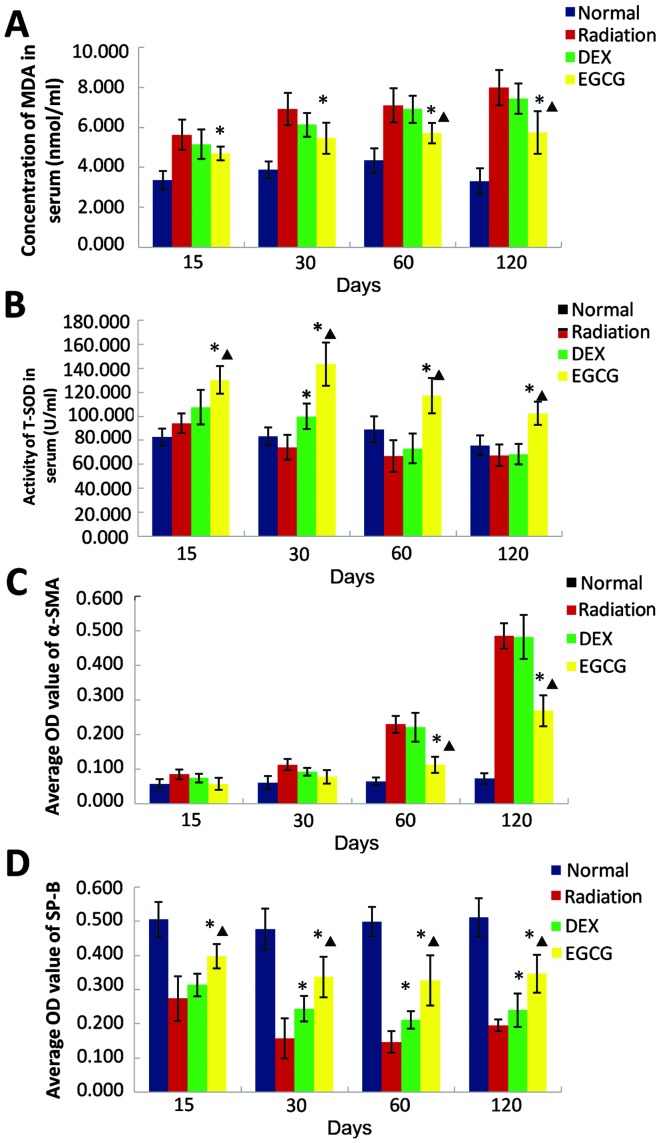
(A) The effect of epigallocatechin-3-gallate (EGCG) on serum malondialdehyde (MDA) concentrations, (B) superoxide dismutase (SOD) activity, (C) α smooth muscle actin (α-SMA) levels and (D) surfactant protein-B (SPB) levels at 15, 30, 60 and 120 days post-irradiation. (A) The MDA concentration in serum was significantly lower (p<0.05) among the EGCG-treated animals compared with that of the dexamethasone (DEX)-treated animals at 60 and 120 days post-irradiation. Conversely, the MDA concentrations of the DEX-treated and untreated animals were comparable at 15, 30, 60 and 120 days post-irradiation. (B) Serum SOD activity was significantly higher (p<0.05) among the EGCG-treated animals compared with the DEX-treated animals at 15, 30, 60 and 120 days post-irradiation. The SOD activity of the latter group was significantly higher (p<0.05) than that of the radiation-only animals at 30 days post-irradiation but similar at 15, 60 and 120 days post-irradiation. (C) The OD value of α-SMA immunohistochemical (IHC) was significantly lower (p<0.05) among EGCG-treated animals than that of the DEX-treated animals at 60 and 120 days post-irradiation. Conversely, the OD value of α-SMA IHC among the DEX-treated animals was comparable to that of the untreated animals at 15, 30, 60 and 120 days post-irradiation (p>0.05). (D) The OD value of SPB IHC was significantly higher (p<0.05) among the EGCG-treated animals relative to the DEX-treated animals at 15, 30, 60 and 120 days post-irradiation. The OD value of SPB IHC among the DEX-treated animals was similar to that of the untreated animals at 15 days post-irradiation but significantly higher (p<0.05) at 30, 60 and 120 days post-irradiation. The bars in the graph are the standard deviations (SDs). Asterisks show significance (p<0.05) compared with the untreated radiation-only animals, and stars show significance (p<0.05) compared with the DEX-treated animals.

We investigated whether EGCG treatment regulates the redox balance post-irradiation. Serum MDA content and SOD activity were measured to assess the oxidative and antioxidant statuses, respectively. The serum MDA concentration was significantly lower (p<0.05) in EGCG-treated animals than DEX-treated animals at 60 and 120 days post-irradiation. The MDA concentration in DEX-treated animals was similar to that of the radiation-only animals at 15, 30, 60 and 120 days post-irradiation (Fig. 4A).

Serum SOD activity was significantly higher (p<0.05) in EGCG-treated animals compared with DEX-treated animals at 15, 30, 60 and 120 days post-irradiation. Levels of SOD activity in the latter group were significantly higher (p<0.05) than those of the radiation-only animals at 30 days post-irradiation but similar at 15, 60 and 120 days post-irradiation (Fig. 4B). These results demonstrated that EGCG treatment modulates redox balance *in vivo.*

### EGCG inhibits (myo)fibroblast proliferation and protects alveolar epithelial type II (AE2) cells from injury

**Figure 5:**
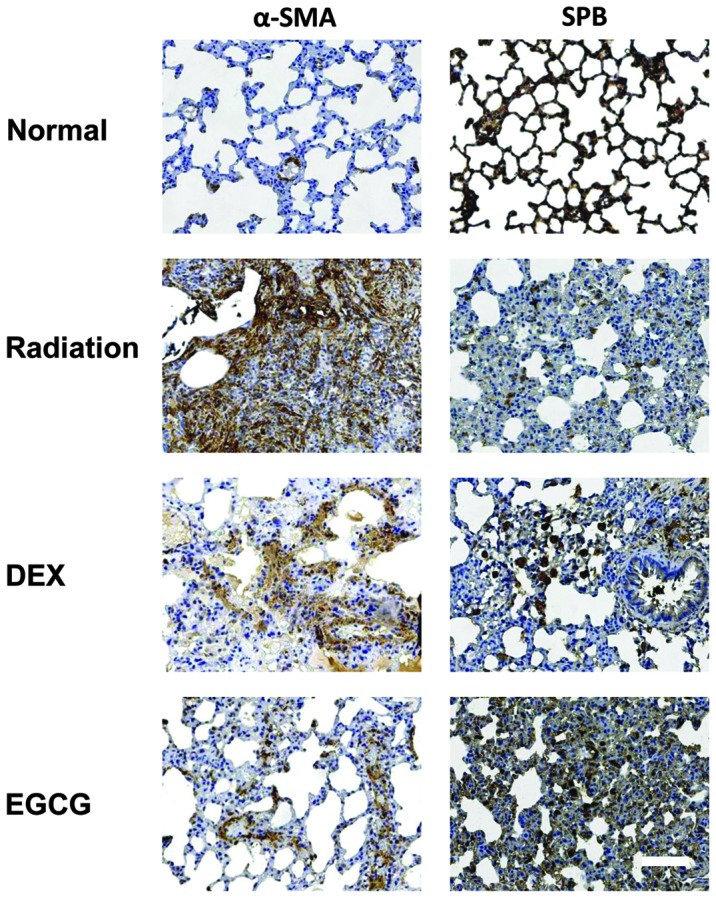
Effect of epigallocatechin-3-gallate (EGCG) on the α smooth muscle actin (α-SMA) and surfactant protein-B (SPB) immunohistochemistry at 120 days post-irradiation. Photomicrographs show the immunohistochemical (IHC) staining with antibodies detecting α-SMA (left column) or SPB (right column) in lung tissue from rats in the non-irradiated (normal), irradiated but untreated (Radiation), dexamethasone (DEX)-treated, and EGCG-treated (EGCG) groups. Myofibroblast accumulation hyperplasia as stained by α-SMA IHC was significantly reduced, and alveolar type II cells (stained by SPB IHC) were significantly increased in EGCG-treated animals. Bar, 100 μm.

The activation and proliferation of (myo)fibroblasts are important contributors to pulmonary fibrosis. Injury to the AE2 cells directly and indirectly contributes to the effects of irradiation-induced pulmonary injury. Therefore, we investigated the effects of EGCG on (myo)fibroblasts and AE2 cells. The expression of α-SMA, a (myo)fibroblast marker, and SPB, an AE2 marker, was investigated using immunohistochemistry on the lung sections. Strong α-SMA expression was observed in the irradiated pulmonary tissues at 120 days post-irradiation. Treatment with EGCG, but not DEX, significantly reduced α-SMA expression (Fig. 5, left column). Conversely, weak SPB expression was observed in the irradiated pulmonary tissues at 120 days post-irradiation. Treatment with EGCG, but not DEX, significantly enhanced SPB expression (Fig. 5, right column).

A morphometric analysis of α-SMA and SPB immunohistochemistry as described in Materials and methods was performed. The OD value for α-SMA immunohistochemistry was significantly lower (p<0.05) in the EGCG-treated animals compared with the DEX-treated animals at 60 and 120 days post-irradiation. The OD value for α-SMA immunohistochemistry in the DEX-treated animals was similar to that of the radiation-only animals at 15, 30, 60 and 120 days post-irradiation (p>0.05; Fig. 4C). Conversely, the OD value for SPB immunohistochemistry was significantly higher (p<0.05) in the EGCG-treated animals compared with the DEX-treated animals at 15, 30, 60 and 120 days post-irradiation. The OD value for SPB immunohistochemistry in the latter group was significantly higher (p<0.05) than that of the radiation-only animals at 30, 60 and 120 days post-irradiation (Fig. 4D). These results confirmed that EGCG inhibits (myo)fibroblast proliferation and protects AE2 cells from injury post-irradiation.

### EGCG regulates serum cytokine levels

**Figure 6:**
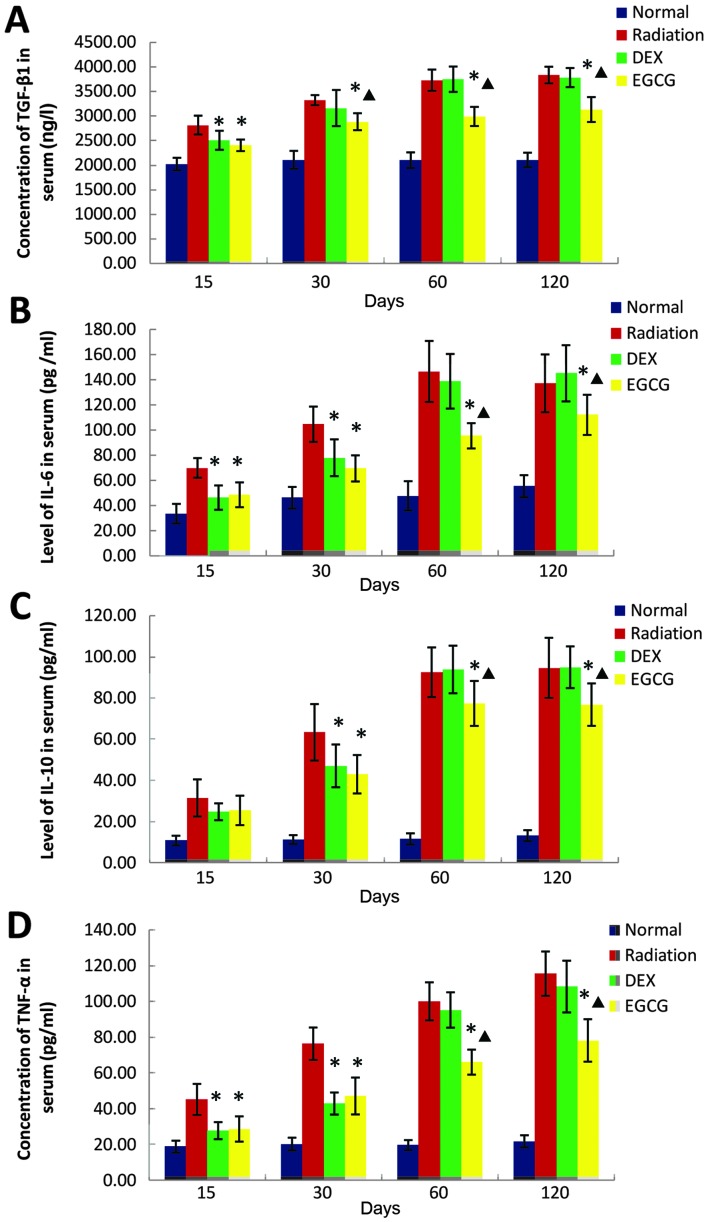
(A) The effect of epigallocatechin-3-gallate (EGCG) on the serum levels of transforming growth factor β1 (TGF-β1), (B) interleukin (IL)-6, (C) IL-10, and (D) tumor necrosis factor α (TNF-α) at 15, 30, 60 and 120 days post-irradiation. (A) Serum TGF-β1 levels were significantly lower (p<0.05) in the EGCG-treated animals compared with those in the dexamethasone (DEX)-treated animals at 30, 60 and 120 days post-irradiation. The serum TGF-β1 levels in the DEX-treated animals were similar to those of the untreated animals at 30, 60 and 120 days post-irradiation. (B–D) The serum levels of IL-6, IL-10, and TNF-α were significantly lower (p<0.05) in the EGCG-treated animals compared with the DEX-treated animals at 60 and 120 days post-irradiation. The serum levels of IL-6 and TNF-α were significantly lower (p<0.05) in the DEX-treated animals compared with those in the untreated animals at 15 and 30 days post-irradiation. The serum levels of IL-10 were significantly lower (p<0.05) in the DEX-treated animals than those in the radiation-only animals at 30 days post-irradiation. The serum levels of IL-6, IL-10, and TNF-α in the DEX-treated animals were similar to those in the radiation-only animals at 60 and 120 days post-irradiation. The bars in each graph are the standard deviations (SDs). Asterisks show significance (p<0.05) compared with the untreated animals and stars show significance (p<0.05) compared with the DEX-treated animals.

We examined whether treatment with EGCG reverses the abnormal expression of cytokines in serum following irradiation. Therefore, we measured the serum levels of TGF-β1 using ELISA as well as IL-6, IL-10, and TNF-α using the BD™ CBA Flex Set. The serum level of TGF-β1 was significantly lower (p<0.05) in the EGCG-treated animals compared with the DEX-treated animals at 30, 60 and 120 days post-irradiation, whereas the level observed in the DEX-treated animals was similar to that of the radiation-only animals at the same time points (Fig. 6A).

The serum levels of IL-6, IL-10 and TNF-α were significantly lower (p<0.05) in the EGCG-treated animals than the DEX-treated animals at 60 and 120 days post-irradiation. The IL-6 and TNF-α levels were significantly lower (p<0.05) in the DEX group compared with the radiation-only animals at 15 and 30 days post-irradiation. IL-10 was significantly reduced (p<0.05) in the DEX-treated animals compared with the radiation-only animals at 30 days post-irradiation. The levels of IL-6, IL-10 and TNF-α in the DEX-treated animals were similar to those of the radiation-only animals at 60 and 120 days post-irradiation (Fig. 6B–D).

### EGCG activates Nrf-2 and its downstream antioxidant enzymes in pulmonary tissues

**Figure 7:**
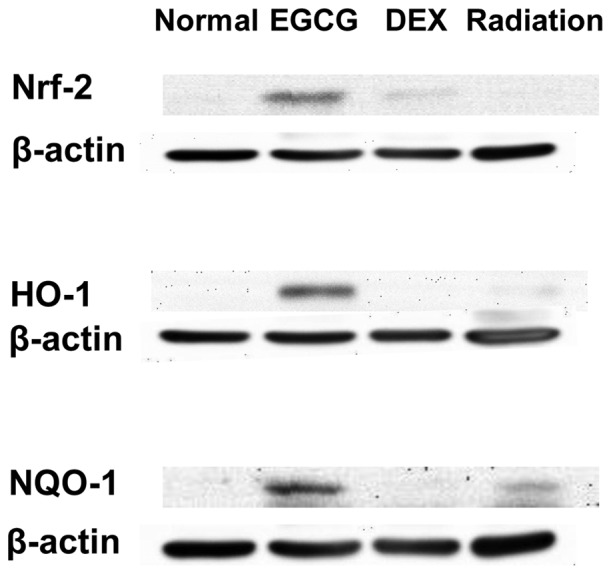
(A) Epigallocatechin-3-gallate (EGCG) activates nuclear transcription factor NF-E2-related factor 2 (Nrf-2), (B) heme oxygenase-1 (HO-1) and (C) NAD(P)H:quinone oxidoreductase-1 (NQO-1) protein expression as detected by western blot analysis of lung tissue extracts at 15 days post-irradiation. Immunoblot analysis revealed that the protein expression of Nrf-2, HO-1, and NQO-1 was strongly activated by EGCG administration, while Nrf-2 was weakly activated by dexamethasone (DEX).

We also examined whether EGCG treatment activates Nrf-2 signaling and its associated antioxidant enzymes. The protein levels of Nrf-2, HO-1 and NQO-1 were assessed by western blot analysis. This analysis revealed that EGCG administration strongly activated Nrf-2, HO-1, and NQO-1 protein levels, whereas DEX administration weakly activated Nrf-2 levels at 15 days post irradiation (Fig. 7).

**Figure 8:**
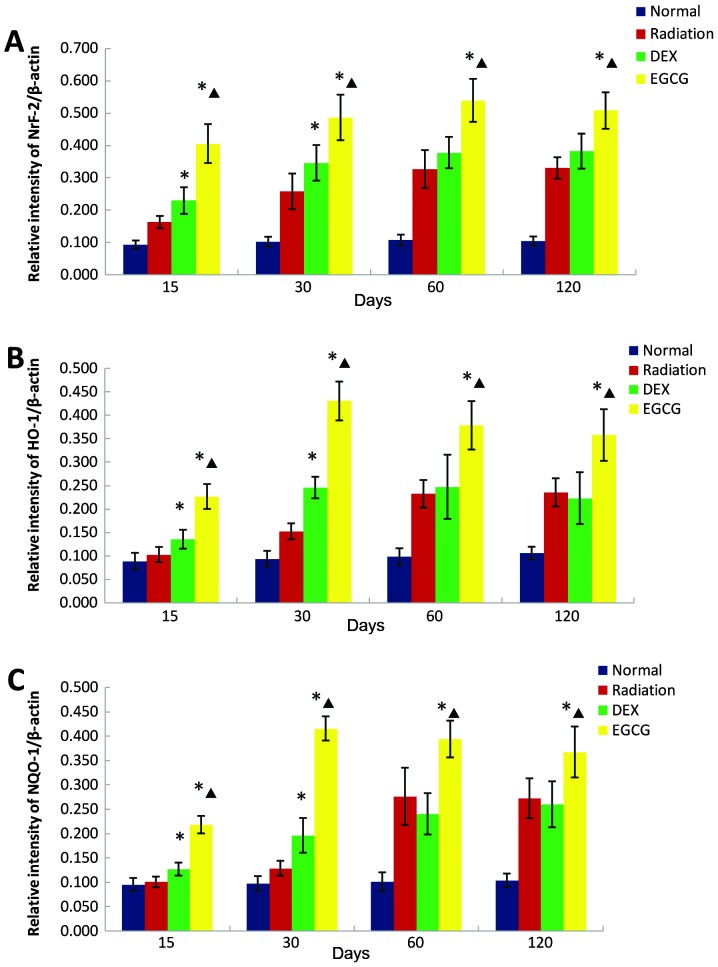
(A) The protein levels of nuclear transcription factor NF-E2-related factor 2 (Nrf-2), (B) heme oxygenase-1 (HO-1) and (C) NAD(P)H:quinone oxidoreductase-1 enzyme (NQO-1) were compared using western blot analysis of lung tissue extracts at 15, 30, 60 and 20 days post-irradiation. The relative protein expression levels of Nrf-2, HO-1, and NQO-1 in lung homogenates were significantly higher (p<0.05) in the epigallocatechin-3-gallate (EGCG)-treated animals than the dexamethasone (DEX)-treated animals at 15, 30, 60 and 120 days post-irradiation. The expression levels of these proteins in the latter group were significantly higher (p<0.05) than those of the radiation-only animals at 15 and 30 days post-irradiation but similar at 60 and 120 days post-irradiation. Experiments were performed with n=6 rats per group, and the western blot analysis OD data were normalized to OD values for β-actin. Data are presented as the means ± standard deviations (SDs). Asterisks show significance (p<0.05) compared with the untreated animals, and stars show significance (p<0.05) compared with the DEX-treated animals.

The protein levels of Nrf-2, HO-1, and NQO-1 were significantly greater (p<0.05) in the pulmonary homogenates from the EGCG-treated animals compared with those of the DEX-treated animals at 15, 30, 60 and 120 days post-irradiation. The Nrf-2, HO-1 and NQO-1 levels of the latter group were significantly higher (p<0.05) than those of the radiation-only animals at 15 and 30 days post-irradiation but similar at 60 and 120 days post-irradiation (Fig. 8).

## Discussion

Results of the present study have shown that irradiation-induced pulmonary fibrosis in rats is principally ameliorated by EGCG administration. EGCG treatment reduced the mortality rate and lung index score, alleviated lung histological damage, reduced collagen deposition, modulated the redox state of serum, inhibited (myo)fibroblast proliferation, protected AE2 cells, and regulated the serum levels of TGF-β1, IL-6, IL-10, and TNF-α. We also showed that EGCG treatment activated Nrf-2 and its downstream antioxidant enzymes HO-1 and NQO-1. The DEX treatment of irradiation-induced pulmonary fibrosis did not produce similarly ameliorative effects. Given these results, we demonstrated that EGCG treatment significantly ameliorates irradiation-induced pulmonary fibrosis. Our data reveal that EGCG has potential for treatments of irradiation-induced pulmonary fibrosis.

Increasing evidence indicates that oxidative stress and ROS contribute directly and indirectly to the formation of irradiation-induced pulmonary fibrosis (19). The ROS-induced activation of inflammatory cells (including macrophages, monocytes, and neutrophils) can cause a positive feedback loop in which an increased expression of a variety of intracellular oxidative enzymes and large amounts of ROS and reactive nitrogen species (RNS) are synthesized and released to remove necrotic tissue (20,21). The dynamics of the oxidant/antioxidant balance in the lung are destroyed, and tissue damage persistently increases. Therefore, any therapeutic intervention that defends against or alleviates oxidant insults may be used to treat irradiation-induced pulmonary fibrosis. Due to its potent antioxidant activity, a variety of animal models have shown that the tea polyphenol EGCG is an effective scavenger of ROS and free radicals with regard to tumors, cardiovascular diseases, and neurological diseases both *in vitro* and *in vivo* (15). The antioxidant activity of EGCG (which likely involves the quenching of ROS, the interception of free radicals, or both) is most likely mediated by an H-atom transfer (HAT) reaction in which intramolecular hydrogen bonding stabilizes the resultant phenoxy radical (22). Previous studies have suggested that EGCG administration inhibits lipopolysaccharide (8) and bleomycin-induced pulmonary fibrosis (11–13). Thus, we hypothesized that scavenging free radicals with EGCG, a natural antioxidant extracted from green tea, inhibits irradiation-induced pulmonary fibrosis.

MDA levels and total SOD activity in serum are important indicators of oxidative stress and the body’s capacity to respond to induced oxidative stress. MDA levels most likely reflect the degree of organic lipid peroxidation, which denotes the severity of damage to cell membranes (23). Conversely, SOD plays a crucial role in the organic oxidative/antioxidant balance. This enzyme can neutralize free radical forms of oxygen, thereby protecting cells from oxidative damage. Moreover, injections of SOD (24) and the SOD mimetic AEOL 10113 (25) have shown protective effects in animal models of radiation-induced fibrosis. Our investigation showed that the serum levels of MDA and inflammatory cytokines decreased, and serum SOD activity increased in DEX-treated animals compared with radiation-only animals at 15 and 30 days post-irradiation. However, these therapeutic benefits ceased with treatment. EGCG-treated rats had greater SOD activity than any other group of rats, including the non-irradiated normal controls, at all time points (15–120 days post-irradiation). This result suggests that EGCG reduced oxidative stress, at least in part, by increasing the systemic production of antioxidant proteins. Overall, our results have demonstrated that EGCG is superior to DEX with regard to increasing the body’s capacity to handle oxidative stress due to ROS/RNS, and these effects were sustained long after treatment was discontinued.

The development of irradiation-induced pulmonary fibrosis is also related to the expression of inflammatory cytokines, which play an important role in creating a positive feedback loop to reinforce the chemotaxis of macrophages and neutrophils and sustaining oxidative stress by supporting the enhanced production of ROS/RNS (26). TGF-β1 is a powerful cytokine that can promote fibroblast proliferation and maturation, thereby accelerating the development of pulmonary fibrosis (27). Wang *et al* demonstrated that TGF-β1 levels were positively correlated with the incidence of radiation-treatment-induced lung injury among patients with lung cancer (28). TNF-α is a driving factor within pro-inflammatory and immunoregulatory networks and is likely involved in the development and progression of radiation-induced pneumonitis (29). In addition, TNF-α stimulates the proliferation of fibroblasts and the secretion of proinflammatory cytokines, including IL-1 and IL-6, from neutrophils and macrophages (30). IL-6 plays an important role in the formation and proliferation of fibrous connective tissue, potentially by increasing collagen aggregation, inhibiting extracellular matrix (ECM) degradation, and stimulating fibroblast proliferation. Previous studies have suggested that IL-6 leads to inflammation and fibrosis associated with hypersensitivity pneumonitis in mice. These results suggest a close relationship in IL-6 and pneumonitis and fibrotic development (31). IL-10 may inhibit monocytes, macrophages, and Th1 cells as well as enhance B-cell immune regulation function (32). In addition, IL-10 is a T-cell-derived cytokine of the Th-2 family that suppresses inflammation by inhibiting numerous pro-inflammatory cytokines (33). Findings of Barbarin *et al* have shown that silica-induced pneumonia and pulmonary fibrosis in mice caused the overexpression of IL-10, thereby contributing to the increased lung damage caused by fibrosis (34).

To investigate the effects of EGCG treatment on systemic inflammation, we measured the serum levels of key inflammatory cytokines including TGF-β1, IL-6, IL-10, and TNF-α. These cytokines were significantly reduced in the EGCG-treated animals compared with the untreated and steroid-treated rats, and this effect lasted for months after treatment ceased. The lower alveolitis score of the EGCG-treated rats also suggested that EGCG reduces the infiltration of inflammatory immune cells. Results of this study are in agreement with those of previous studies that have shown significant anti-inflammatory effects from the administration of EGCG (10,35,36).

This study also investigated the protective effect of EGCG on AE2 cells. AE2 and vascular endothelial cells are the two major targets of radiation-induced lung injury from inflammation and oxidative stress. Results of the SPB staining analysis in the lung revealed that EGCG-treated rats showed a more normalized distribution of AE2 cells in the parenchyma compared with radiation-only and DEX-treated rats. AE2 cells were abundant in alveolar walls of the lung tissues of the EGCG-treated rats, although no evidence of dysplasia was found. These results suggest that EGCG protected parenchymal and AE2 cells from free radical damage. In addition, the myofibroblast proliferation in the lung (as demonstrated by α-SMA staining) observed in the radiation-only and DEX-treated groups was significantly reduced in rats treated with EGCG at 60 and 120 days after irradiation, suggesting that EGCG-inhibited pulmonary fibrosis partially inhibits myofibroblast transformation and proliferation.

We also examined whether EGCG improves the ability of the endogenous oxidative stress response system by activating Nrf2 and its downstream antioxidant enzymes. Sriram *et al* investigated the protective effects of EGCG in a bleomycin-induced acute lung injury animal model and presented the first evidence that EGCG protection against lung injury is associated with the Nrf2-based activation of the oxidative stress response (12). Nrf2 plays a critical role in the regulation of the major antioxidant enzymes HO-1 and NQO-1. Sahin *et al* (37) reported that EGCG significantly reduced the production of peroxides and the subsequent peroxidation of lipids by enhancing the expression of antioxidant enzymes (e.g., SOD, CAT, and GPx) to improve the oxidative stress response. Those authors also showed that EGCG may increase the downstream expression of other antioxidant enzymes by activating Nrf2 and HO-1, thereby regulating oxidative stress. Our western blot analysis results revealed that EGCG significantly enhanced the expression levels of Nrf-2, HO-1, and NQO-1 in rat lung tissues compared with radiation-only and DEX-treated rats, thereby confirming the results of Sriram *et al* and Sahin *et al* (12,37) as well as supporting our hypothesis that EGCG relieves oxidative stress by activating Nrf2 and its associated antioxidant enzymes.

Since glucocorticosteroids are commonly used to treat irradiation-induced pulmonary fibrosis and other forms of lung fibrosis in humans, DEX was selected as a baseline to compare the efficacy of EGCG using various measures of lung inflammation, the oxidative stress response, and fibrosis. A marginal effectiveness was achieved with DEX therapy at 15 and 30 days post-irradiation; however, these improvements ceased following discontinuing steroid therapy (i.e., at 60 and 120 days post-irradiation). The measures of lung inflammation, oxidative stress, and fibrosis among the DEX-treated rats were similar to those of the radiation-only group at 60 and 120 days post-irradiation, suggesting a lack of persistent therapeutic effects. Conversely, our results demonstrate that EGCG was superior to glucocorticoids with regard to reducing inflammation, fibrosis, and oxidative stress during the treatment period (which ended at 30 days post-irradiation). In addition, the therapeutic effects of EGCG were sustained even after treatment ceased, unlike the steroid treatment.

Collectively, results of the present study have shown that EGCG treatment provides strong, persistent antioxidant, anti-inflammatory, and anti-proliferative effects that protect against irradiation-induced pulmonary fibrosis in rats. The findings suggest that these effects are mediated by inhibiting pro-inflammatory immune cells from infiltrating alveoli and lung parenchyma, suppressing the expression of pro-inflammatory factors, and inhibiting the synthesis and secretion of ROS/RNS-free radicals that cause extensive oxidative damage to parenchymal cells. EGCG also inhibited myofibroblast proliferation and AE2 cell dysplasia, presumably by suppressing the secretion of TGF-β1. Of note, the findings demonstrate that these effects reduced the rates of morbidity and mortality compared with those among the rats in the DEX group.

## Abbreviations

EGCG: epigallocatechin-3-gallate
DEX: dexamethasone
MDA: malondialdehyde
SOD: superoxide dismutase
TGF-β1: transforming growth factor β1
IL-6: interleukin-6
IL-10: interleukin-10
TNF-α: tumor necrosis factor α
SPB: surfactant protein-B
α-SMA: α smooth muscle actin
Nrf-2: nuclear transcription factor NF-E2-related factor 2
HO-1: heme oxygenase-1 enzyme
NQO-1: NAD(P)H:quinone oxidoreductase-1 enzyme
AE2: alveolar epithelial type II
